# The Change of Practitioner’s Self-Efficacy in Triple P Professional Training: Moderating Role of Practitioner Characteristics, Country, and Delivery Mode

**DOI:** 10.1007/s10826-023-02568-2

**Published:** 2023-04-15

**Authors:** Matthew R. Sanders, Nam-Phuong T. Hoang, Ruby J. Gerrish, Alan Ralph, Jenna McWilliam

**Affiliations:** 1 Parenting and Family Support Centre, School of Psychology, The University of Queensland, Brisbane, QLD Australia; 2 Triple P International, Indooroopilly, QLD Australia

**Keywords:** Professional training, Self-efficacy, Videoconference, Triple P

## Abstract

Two studies examined the change in self-efficacy of practitioners after attending Triple P training and the moderators that affect training outcomes. Study 1 used a large multidisciplinary sample of health, education, and welfare practitioners (*N* = 37,235) came from 30 countries around the world, which all participate in a Triple P professional training course during 2012–2019. This study assessed practitioners’ overall self-efficacy and their consultation skills efficacy prior to training, immediately following training, and at six- to eight-weeks follow-up. Participants reported significant improvements of their overall self-efficacy and their consultation skills self-efficacy. There were significantly small differences based on practitioners’ gender, disciplines, education levels, and country location. Study 2 examined the training outcomes of videoconference-based training (following the COVID-19 pandemic) compared to in-person training (*N* = 6867). No significant differences were found between videoconference and in-person training on any outcome measure. Implications for the global dissemination of evidence-based parenting programs as part of a comprehensive public health response to COVID-19 was discussed.

## Highlights

- This manuscript examined the change of practitioners’ self-efficacy throughout the course of Triple P (Positive Parenting Program) system professional training via the presentation of two studies. Study 1 used a large multidisciplinary sample of practitioners (*N* = 37,235) from 30 countries worldwide. Study 2 examined the training outcomes on practitioners’ self-efficacy comparing between videoconference-based training (following COVID-19 pandemic) and in-person training (*N* = 6867).
- In both studies, practitioners reported a significant change in their self-efficacy to deliver the Triple P program with their clients.
- Significant but small differences were observed between gender, educational levels, disciplines and countries of delivering (English speaking or non-English speaking).
- No significant differences were found between videoconference and in-person training on any outcome measure.

Self-efficacy is an important psychological construct that has been widely studied in the fields of psychology, education, and health care. It refers to an individual’s belief in their ability to mobilize cognitive resources, motivation, and actions needed to achieve a task demand (Bandura, 1977). According to Bandura (1989), self-efficacy is more than a passive estimate of future performance; it involves a generative capacity for orchestrating resources and subskills. Self-efficacy is often seen as an important predictor of performance and persistence, as individuals with higher levels of self-efficacy are more likely to engage in tasks, persist in the face of challenges, and achieve better outcomes.

In health and clinical training contexts, self-efficacy has long been recognized as a measure of progress (Mullen et al., 2015). This factor is identified as a crucial component of the practitioner characteristics dimension. Practitioners with a higher level of self-efficacy are more likely to initiate program use and to persist despite obstacles. They are also able to utilize their skills in real-life settings and directly influence the quality of their services (Ma et al., 2023). Self-efficacy has been included in multiple sustainability or implementation frameworks with many studies demonstrating its role in predicting program use (Damschroder et al., 2020; Mazzucchelli & Ralph, 2019). In turn, practitioners’ self-efficacy and motivation to use the program again will improve once the intervention is successfully implemented (Turner et al., 2011).

Considering the importance of self-efficacy in post-training contexts, most health professions programs emphasize both theoretical foundations and clinical practice components (Latorre et al., 2021; Mohamed & Fashafsheh, 2019). The objective is to support trainees in building the self-efficacy necessary to guide judgment and practice outside of the training context. This is done by providing opportunities for them to practice the skills during training.

## Triple P Professional Training Model

The Triple P-Positive Parenting Program (Sanders, 2012) training system is no exception. Triple P is a comprehensive, multi-level system of evidence-based parenting support based on a population approach (Sanders & Mazzucchelli, 2017). The program includes multiple variants targeting different populations (e.g. Group, Standard, Primary Care), different levels of intensity (e.g. low intensity universal seminars and more intensive multi session group programs), different age groups (infancy through to adolescence), and modes of delivery (in person individual, group, self-directed and online). To become accredited practitioner of the Triple P, practitioners need to attend a training workshop and complete a competency assessment 6–8 weeks after training. The current training courses run between 2–5 days depending on the level and variant of Triple P.

In order to support practitioners to become independent problem solvers and being able to apply their learned skills in their usual practice context, the training system of Triple P adopted an active skills training approach that emphasizes self-directed learning, personal goal setting, self-evaluation, and problem-solving (Mazzucchelli & Ralph, 2019; Turner et al., 2011). Within Triple P workshops, practitioners learn to become independent problem solvers via the promotion of self-regulatory capacity and self-reflective practice using various instructional modes (e.g., goal setting, didactic presentations, video demonstrations, role playing of skills and self-evaluation). One important aim of the training is to improve practitioners’ self-efficacy or their beliefs in the ability to manage one’s behavior and emotions to achieve specific goals (Karoly, 1993). Thus, as part of the quality assurance process established for Triple P, data on practitioner self-efficacy are routinely collected prior to, and at the end of the Triple P training and accreditation.

Over the years, there has been increasing evidence to show that the Triple P professional training is effective in increasing practitioners’ self-efficacy in consultation skills and knowledge. In an Australian-based study, Sanders et al. (2003) evaluated the training effectiveness of Primary Care Triple P (Level 3). They found that general medical practitioners (*N* = 331) reported a significant improvement in parent consultation skills post-training. Shapiro et al. (2008) examined the feasibility of a large-scale Triple P training program to existing service providers (*N* = 448) in the United States. Results demonstrated significant improvement in practitioner self-efficacy and competence in the delivery of Triple P, with participants reporting a high degree of self-efficacy and self-reported skills in delivering parent consultations. Sethi et al. (2014) evaluated the effectiveness of Level 4 training between 2007 and 2012 (*N* = 5109) and found consistent improvements of practitioners’ perceived adequacy of knowledge and skills at post-intervention and at follow-up. Similar findings were reported by Ralph and Dittman (2018), in an evaluation of 2961 training courses between 2012 and 2016. In this evaluation, practitioners’ adequacy, self-efficacy and perceived skills were reported to consistently increase from pre to post-training and at follow-up (Ralph & Dittman, 2018).

Though evidence has shown that the Triple P professional training system was effective in improving practitioners’ self-efficacy, it was unknown if these effects can be upheld when the differences in training levels and practitioners’ characteristics are accounted for. Over the years, with the recognition that to reduce the prevalence of social, emotional and behavioral problems in children at a population level, a well-trained multidisciplinary workforce is required, Triple P has trained a large workforce that extend outside of mental health (Beidas et al., 2009; Sanders et al., 2018). This workforce includes professionals from different disciplines with different academic qualifications (psychologists, social workers, medical practitioners, nurses and other allied health professionals and early childhood educators, educators and family support workers) who work in various settings such as primary health care, mental health, primary health care, hospital settings, early childhood, schools and in the social care/ welfare sectors to implement evidence-based parenting interventions (EBPIs). This workforce is also diverse in term of prior experience, and personal and organizational allegiance to using evidence-based programs (Brookman-Frazee et al., 2018; Hodge et al., 2017). All of those variables might influence practitioners' self-efficacy throughout the training program. Indeed, studies on self-efficacy has shown that one’s self-perceived capacity is impacted by many learners’ characteristics. For example, the self-efficacy literature indicates that there are potential differences between genders when it comes to their reported self-efficacy with male are more likely to rate their self-efficacy higher yet females usually demonstrate a greater improvement as they are more likely to structure their environment for optimal learning outcomes (Pajares, 2002). Personal experience with similar tasks or similar training also helps with the development of performance strategies, which may alter perceived task demands or leaners’ coping ability (Gist & Mitchell, 1992).

It is also relevant to consider whether the training was conducted in English-speaking countries or in non-English-speaking countries. Triple P was originally developed in Australia and initial training was delivered in English-speaking countries. Gradually, as the demand for training increased, the program was also translated and delivered in other non-English speaking countries. When delivering the program in a country that speak languages other than English, Triple P takes an active approach to make sure that the program is appropriate to serve the culture and language of the target population. The training program is delivered by a trainer who speaks the local language, and the materials are translated where possible. A flexible training approach is used during training to encourage practitioners to adhere to the program’s core elements while also working collaboratively with parents, responding to the families’ needs within the context of cultural, social, and environmental constraints (Sanders & Mazzucchelli, 2017). Such flexibility provides practitioners opportunities to use culturally appropriate examples, customize homework, and structure their delivery in ways that fit local parents. With this “build-in” strategic flexibility, the program is able to maintain the core concepts and procedures while achieving a high level of cultural sensitivity at the lowest cost (Sanders & Mazzucchelli, 2017). Despite this integrated, innovative strategy, it is important to note that most training courses at the moment are still being delivered in English. The majority of non-English-speaking practitioners thus still face language barriers as well as differences in thinking and values. The differences might influence participants’ perceptions of the program’s appropriateness and, in turn, their ability to effectively utilize the strategies once they are trained.

The delivery mode is also another factor that need to be considered when it comes to the practitioners’ self-efficacy. Prior to the COVID-19 pandemic, most clinical and counselor training programs – Triple P included - have traditionally been hesitant to incorporate online learning into their curriculum (Benshoff & Gibbons, 2011). Such common hesitancy has arisen concerning whether online education is appropriate for clinical professions in which the development of basic counselor-client relationship skills is an integral part of the training (Murphy et al., 2008), or whether the clinical techniques can be efficiently delivered online (Wantz et al., 2003). The COVID-19 pandemic however had a dramatic impact on many organizations including training organizations that relied on in-person service delivery. Demand for training was still present, although the feasibility of in-person training decreased substantially as many countries went into lockdown and social distancing advice prevented in-person groups. Triple P International (TPI) (the licensed purveyor organization responsible for Triple P dissemination) transitioned immediately to offering training courses via videoconference technology. The use of videoconferencing for skills-based training required substantial effort from both trainers and practitioners to adapt to the online delivery system. Trainers had to learn how to join groups in breakout rooms to provide feedback, and practitioners needed to learn how to do role plays without being physically present. Given all the new technical requirements attached to videoconferencing and probable reduction of hand-on support of the trainer during role-plays, it was unknown if this new modality of training could achieve the same effect to improve practitioners’ self-efficacy as the well-established in-person training.

## The Present Study

This paper aims to examine the change of practitioners’ self-efficacy over the course of Triple P professional training program and several moderators that might impact such training outcome. Our primary research questions were as follows:

1. Does practitioners’ self-efficacy improve between pre-training, post training and at 6–8-week follow-up of attending a Triple P training program?
2. Does practitioners’ self-efficacy improvement vary across genders, educational levels, training backgrounds, countries (English vs non-English) and modality of training (in-person vs videoconferencing)?

These research questions will be examined via the presentation of two studies. Study 1 examines training outcome data of practitioners who completed a level of Triple P training between 2012–2019. This study explored the effects of different practitioners’ individual variables on training outcomes such as professionals’ background and educational qualifications. Study 2 displays data from practitioners who completed various levels of Triple P training between January 2020 and December 2020. This study examined the differences in training outcomes between in-person (traditional model) courses and using videoconferencing technology such as Zoom. Within this study, we also sought to gain an insight into the logistical challenges that arise due to transitioning from in-person to videoconference based training.

Based on prior research, we expected a significant improvement in practitioner self-efficacy post training. We predicted that overall, there would be a similar pattern of improvement in training outcomes across country groups, practitioners’ training background, education levels, and gender. We anticipated there might be differences in training outcomes across countries, such that improvements would be larger among English-speaking countries compared to non-English-speaking countries, but there would be no differences found among practitioners in terms of their improvements across disciplines, education levels, and gender. We also expected that the pattern of improvement would be similar between in-person training and videoconference training.

## Method

### Participants

#### Study 1

Participants were 37,235 practitioners who completed a level of Triple P training between 2012–2019, organized by TPI. Data was collected as a routine evaluation of training workshop by TPI. Most practitioners were female (89.8%) and the majority of practitioners in our sample got trained at Level 3 (41.3%) or Level 4 (51.8%) with a small number of participants participated in Level 2 (4.9%) and Level 5 (2.1%). It is important to note that partitioners can get trained at multiple levels of Triple P program over the years. For this analysis, only their first training with Triple P during the period of 2012 and 2019 were included. There are different pathways in which participants enrolled in the program, but the majority of participants were sent to get trained by their organizations including government (40.3%), non-government (41.6%) and other private sector/ research/ university (10.3%). The training was delivered in 30 countries with practitioners from various educational levels and professional backgrounds (See Table 1 for further details).

**Table 1:** Study 1 means and standard deviations of PCSC subscales at pre-training, post-training, and follow-up by moderators

Variable			Overall self-efficacy						Consultation skills self-efficacy					
			Pre		Post		Follow-up		Pre		Post		Follow-up	
	*n*	*%*	*M*	*SD*	*M*	*SD*	*M*	*SD*	*M*	*SD*	*M*	*SD*	*M*	*SD*
*Overall training outcomes*	37,235	100	4.29	1.29	5.54	0.88	5.98	0.75	4.36	1.11	5.6	0.78	5.95	0.66
*Training outcomes by Moderators*														
*Level of training*														
Level 2	1806		3.89	1.51	5.49	0.94	5.92	0.77	4.43	1.18	5.57	0.83	5.88	0.66
Level 3	15,379		4.32	1.24	5.45	0.89	5.99	0.75	4.24	1.13	5.51	0.79	5.95	0.64
Level 4	19,275		4.28	1.31	5.49	0.94	5.96	0.76	4.43	1.09	5.66	0.77	5.95	0.67
Level 5	775		5.08	1.15	5.45	0.89	6.14	0.70	4.96	0.88	5.92	0.66	6.10	0.56
*Gender*														
Female	33,470		4.28	1.29	5.54	0.88	5.98	0.75	4.36	1.11	5.60	0.78	5.95	0.66
Male	3423		4.44	1.30	5.61	0.89	6.02	0.76	4.47	1.12	5.64	0.79	5.97	0.64
*Education levels*														
Bachelor	14,545		4.22	1.29	5.51	0.85	5.96	0.74	4.33	1.08	5.59	0.75	5.94	0.63
Doctorate	970		4.58	1.38	5.85	0.85	6.14	0.73	4.54	1.18	5.80	0.78	6.08	0.65
Master	9496		4.5	1.3	5.76	0.84	6.13	0.72	4.55	1.07	5.79	0.71	6.08	0.60
High School	2599		4	1.31	5.25	0.97	5.84	0.84	4.09	1.26	5.34	0.93	5.82	0.77
Tertiary^a^	7095		4.27	1.24	5.46	0.86	5.92	0.74	4.32	1.10	5.53	0.80	5.90	0.66
*Disciplines*														
Allied health and correction services	1040		4.01	1.33	5.49	0.86	5.92	0.75	4.14	1.17	5.58	0.78	5.93	0.62
Teachers/Educators	9285		4.33	1.26	5.55	0.88	6.01	0.75	4.36	1.11	5.60	0.79	5.97	0.66
Mental health^b^ worker	13,698		4.44	1.26	5.66	0.85	6.06	0.73	4.56	1.02	5.73	0.72	6.03	0.61
Medical personnel	3578		3.92	1.26	5.27	0.83	5.74	0.73	3.89	1.07	5.31	0.76	5.72	0.65
Others	6139		4.18	1.36	5.53	0.9	6.01	0.76	4.29	1.19	5.61	0.81	5.98	0.66
*Country groups*^*c*^														
English speaking	30,321		4.24	1.31	5.59	0.88	6.03	0.75	4.37	1.13	5.67	0.76	6.01	0.63
Non-English speaking	6914		4.51	1.18	5.33	0.83	5.74	0.74	4.32	1.04	5.28	0.81	5.68	0.71

^a^Tertiary includes Trade/Diploma

^b^Mental Health Worker included Psychologists, Social Workers and Counselors

^c^The English-speaking countries included England, Scotland, Wales, and Ireland. The non-English speaking countries included Africa, Asia (China, Hong Kong, Japan, Singapore and others), Latin America (Argentina, Chile, Colombia, Costa Rica, Mexico) and non-English speaking European Union (Belgium, France, the Netherlands and Sweden, Dutch Caribbean, Denmark, Curacao, Ireland, and Switzerland)

#### Study 2

Participants were 6867 practitioners who completed various levels of the Triple P training between January 2020 and December 2020. Among these, 5138 practitioners participated in in-person training and 1684 practitioners completed training via videoconference (See Table 4). Among all 6867 trained practitioners, 6213 (90.5%) of them later got accredited as certified Triple P practitioners.

### Measures

In both studies, the same measures were used to evaluate the training outcomes including:

#### Demographics

Demographics data were collected including practitioners’ gender, their highest qualification, their areas of training and also their employment details.

#### Parent Consultation Skills Checklist (PCSC)

The PCSC (Turner & Sanders, 1996) assesses practitioners’ self-efficacy in conducting parenting consultations with parents. The Checklist comprises 22 items. Factor analysis was done with Study 1’s sample indicated that the scale fit data well (*X*^*2*^ = 3745.04, *df* = 3723.04, *p* < 0.05; *CFI* = 0.96, *RSMEA* = 0.055) and the first two items of the scale, assessing practitioners’ adequacy *(“Do you feel adequately trained to conduct parent groups about child behavior?”)* and confidence *(“How confident are you in conducting parent groups about child behavior?”)* loaded onto one factor. These two items were then combined as the Overall Self-Efficacy subscale. The remaining 20 items assess self-rated proficiency in using a range of consultation skills (e.g., *“Demonstrating the use of specific positive parenting skills”*). Responses are scored on a scale of 1 (*not at all proficient*) to 7 (*extremely proficient, no assistance required*). These twenty items loaded onto a second factor, forming the Consultation Skills Self-Efficacy subscale. Both Overall-Self-Efficacy (pre-training *α* = 0.89; post-training *α* = 0.83; and follow-up *α* = 0.82) and Consultation skills self-efficacy subscales (pre-training *α* = 0.97; post-training *α* = 0.97; and follow-up *α* = 0.97) demonstrated good to excellent internal consistency reliabilities with this study sample. The previous study using PCSC 17-item version indicated an overall Cronbach’s at α = 0.96, and an average inter-item correlation of 0.61 (Sethi et al., 2014). There is a strong correlation between the items when the correlation is between 0.48 and 0.76 (Briggs & Cheek, 1986).

### Procedure

Practitioners completed the PCSC at three time points: (1) at the commencement of the course (T1, pre-training), (2) immediately at the completion of the course (T2, post-training), and (3) following accreditation six- to eight-weeks after the initial training (T3, follow-up). Standardized training protocols were administered at each international site and each training event. Different languages were used to deliver training depending on the location (including English, Spanish, French, Dutch, German, Japanese and Cantonese).

## Results

Data was analyzed using the R package WRS2 (Mair & Wilcox, 2019). Similar statistical procedures were applied to the two data sets. The effect of time (pre-training, post-training, and follow-up) was calculated with the WRS2 *rmanova* function, followed by post hoc *rmmpc*, to calculate change across time. In addition, potential interactions between time and the moderators were calculated with the *bwtrim* function of WRS2 package, followed by post hoc analyses with *sppbi* functions to calculate differences in rates of improvement across groups. The Explanatory measure of Effect size was also calculated to estimate the magnitude of effect. Explanatory measure of effect size applies the same rules of thumb as for Cohen’s d of which 0.2, 0.5, and 0.8 correspond to small, medium, and large effects. Due to the very large sample size, we conservatively set the *p* value at 0.0001.

### Attrition and Missing Data

For both studies, 100% of participants attended at least one training module and the majority of them provided post assessment data (Study 1: 97.3%; Study 2: 95.4%). For the follow-up assessment, 86.6% (Study1) and 86.7% (Study 2) continue to provide their data. For both studies, the drop-out rate was under 13.4%. To avoid reporter bias, all practitioners were retained for analysis for both Study 1 and Study 2. Maximum likelihood estimation method was used to handle missing data.

The exploration of accreditation pointed out that most participants (86% for Study 1 and 90.5% for Study 2) went on and get accredited after training. Comparison between the groups that got accredited and those who did not get accreditation for Study 1 sample indicated statistical significant difference between two groups in terms of their overall self-efficacy *F*(1, 4130.96) = 3.6173, *p* < 0.001 but not for their consultation skills self-efficacy *F*(1,4240) = 2.437; *p* > 0.0001. The effect size of differences however is negligible at 0.04 [0.02–0.08]. Outcomes of Study 2 indicated no significant differences between the accredited and non-accredited group regarding their overall self-efficacy *F*(1520.74) = 6.838; *p* > 0.0001 and their consultation skills self-efficacy *F*(1493.38) = 2.189, *p* > 0.001.

## Study 1

### Overall Training Outcomes

Robust repeated measures ANOVA indicated significant improvements in practitioners’ overall self-efficacy subscale from pre-training to post training *F*(1,22340) = 30701.3 *p* < *0*.0001, and from pre-training to follow-up *F*(1.59, 54407.52) = 81192.62, *p* < 0.0001 from pre training to post training and from pre training to follow-up *F*(1,22340) = 56929.28 *p* < *0*.0001. Similar outcomes were found for the practitioners’ consultation skills self-efficacy with significant improvement from pre-training to post training *F*(1,22340 = 53237.69)*, p* < *0*.0001 and from pre training to follow-up *F*(1,22340) = 74260.28*, p* < *0*.0001.

Post-hoc analysis was carried out to calculate the differences in training outcomes across levels of training. Significant differences between Level of training were found with practitioners of level 5 reported highest rate of overall self-efficacy and consultation skills self-efficacy across all time point (Table 1) yet, practitioners who got trained in Level 3 demonstrated the largest improvement both at post training and follow-up followed by those got trained in Level 4 (Table 3). The differences however were negligible with the Exploratory measure of effect size was 0.06 [0.02–0.009] at post training and 0.07 [0.04–0.11] at follow-up. No further control for training levels was applied.

## Training Outcomes by Practitioners’ Characteristics

### Gender

A significant time by group interaction was found for gender on practitioners’ overall self-efficacy at post training and at follow-up (Table 2). Significant differences were also found for practitioners’ consultation skills self-efficacy at post-training and at follow-up (Table 3).

**Table 2:** Study 1 post-hoc analysis of the effects of time and individual differences on practitioners’ overall self-efficacy

		M_change 1_(SD)	*F*(df1, df2) *p* Effect size *[95% CI]*	M_change 2_(SD)	*F*(df1, df2) *p* Effect size *[95% CI]*
*Overall Self-Efficacy*		1.31 (0.90)	*F(*1,22340*)* = 30701.3 *p* < 0.0001	1.69 (0.95)	*F(*1,22340*)* = 56929.28 *p* < 0.0001
*Level of training*	Level 2	1.60 (1.45)	*F*(3,3888.86) = 148.90 *p* < 0.0001 0.06 [0.02–0.09]	2.04 (1.54)	*F*(3, 3793.59) = 113.37 *p* < 0.0001 0.07 [0.04–0.11]
	Level 3	1.13 (1.20)		1.67 (1.28)	
	Level 4	1.33 (1.21)		1.69 (1.29)	
	Level 5	0.83 (1.04)		1.07 (1.11)	
*Gender*	Female	1.26 (1.22)	*F*(1 24440.95) = 14.80 *p* < 0.0001 0.05 [0.06–0.08]	1.7 (1.30)	*F*(1, 24134.71) = 20.29 *p p* < 0.0001 0.05 [0.04–0.06]
	Male	1.16 (1.20)		1.58 (1.28)	
*Education levels*	Bachelor	1.30 (1.25)	*F*(4, 3059.89) = 6.496 *p* < 0.0001 0.21 [0.20–0.22]	1.75 (1.31)	*F*(4, 3031.23) = 14.61 *p* < 0.0001 0.15 [0.14–0.16]
	Doctorate	1.28 (1.16)		1.56 (1.23)	
	Master	1.26 (1.19)		1.63 (1.29)	
	Highschool	1.25 (1.30)		1.84 (1.38)	
	Tertiary	1.19 (1.19)		1.65 (1.28)	
*Disciplines*	Allied Professional	1.48 (1.31)	*F*(4, 2993.76) = 22.27 *p* < 0.0001 0.10 [0.05–0.15]	1.91 (1.33)	*F*(4, 3027.49) = 31.20 *p* < 0.0001 0.08 [0.04–0.13]
	Educator	1.22 (1.22)		1.67 (1.30)	
	Mental Health Worker	1.22 (1.19)		1.62 (1.26)	
	Medical Personnel	1.35 (1.22)		1.82 (1.28)	
	Others	1.35 (1.28)		1.83 (1.37)	
*Country groups*	Non-English speaking	0.82 (1.07)	*F*(1,6326.80) = 1095.79 *p* < 0.0001 0.00 [0.00–0.02]	1.23 (1.14)	*F*(1, 6264.81) = 1341.79 *p* < 0.0001 0.00 [0.00–0.02]
	English speaking	1.35 (1.23)		1.79 (1.31)	

*M*_*change 1*_ Mean change from Pre-training to Post training, *M*_*change 2*_ Mean change from Pre-training to Follow-up, *Effect size* Explanatory measure of effect size calculated with WRS2

**Table 3:** Study 1 post-hoc analysis of the effects of time and individual differences on consultation skills self-efficacy

		M_change 1_(SD)	*F*(df1, df2) *p* Effect size *[95% CI]*	M_change 2_(SD)	*F*(df1, df2) *p* Effect size *[95% CI]*
*Overall Consultation Skills Self-Efficacy*		1.24 (0.90)	*F(*1,22340 = 53237.69*) p* < 0.0001	1.59 (0.90)	*F(*1,22340*)* = 74260.28 *p* < 0.0001
*Level of training*	Level 2	1.15 (1.00)	*F*(3, 3771.07) = 37.65 *p* < 0.0001 0.09 [0.05–0.12]	1.46 (1.14)	*F*(3, 3750.00) = 151.41 *p* < 0.0001 0.04 [0.00–0.07]
	Level 3	1.27 (1.01)		1.71 (1.10)	
	Level 4	1.23 (0.94)		1.52 (1.03)	
	Level 5	0.95 (0.75)		1.14 (0.83)	
*Gender*	Female	1.25 (0.97)	*F*(1, 24292.00) = 18.58 *p* < 0.0001 0.05 [0.04–0.06]	1.60 (1.07)	*F*(1, 24164.24) = 34.45 *p* < 0.0001 0.41 [0.03–0.05]
	Male	1.17 (0.95)		1.50 (1.05)	
*Education levels*	Bachelor	1.27 (0.97)	*F*(4, 3027.02) = 2.68 *p* < 0.0001 0.15 [0.14–0.16]	1.62 (1.05)	*F*(4, 3020.80) = 15.59 *p* < 0.0001 0.11 [0.11–0.13]
	Doctorate	1.26 (0.93)		1.54 (1.01)	
	Master	1.24 (0.92)		1.53 (1.02)	
	Highschool	1.24 (1.15)		1.73 (1.24)	
	Tertiary	1.21 (0.97)		1.58 (1.09)	
*Disciplines*	Allied Professional	1.44 (1.06)	*F*(4, 2956.52) = 54.498 *p* < 0.0001 0.06 [0.02–0.11]	1.79 (1.14)	*F*(4, 2950.60) = 89.21 *p* < 0.0001 0.05 [0.015–0.11]
	Educator	1.24 (0.99)		1.61 (1.09)	
	Mental Health Worker	1.17 (0.89)		1.48 (0.98)	
	Medical Personnel	1.42 (0.98)		1.83 (1.07)	
	Others	1.32 (1.05)		1.69 (1.16)	
*Country groups*	Non-English speaking	0.96 (0.85)	*F*(1,6338.07) = 649.15 *p* < 0.0001 0.16 [0.14–0.18]	1.37 (0.96)	*F*(1, 6318.01) = 367.25 *p* < 0.0001 0.13 [0.12–0.15]
	English speaking	1.3 (0.98)		1.64 (1.08)	

*M*_*change 1*_ Mean change from Pre-training to Post training, *M*_*change 2*_ Mean change from Pre-training to Follow-up, *Effect size* Explanatory measure of effect size calculated with WRS2

Post-hoc analysis indicated that male practitioners reported higher self-efficacy scores than female practitioners at all three time points (Table 1). However, female practitioners reported greater overall improvement in self-efficacy than male practitioners from pre-training to post-training and pre-training to follow-up (Table 2). Same pattern was occurred for practitioners’ consultation skills self-efficacy, with male practitioners reporting higher self-efficacy in applying the consultation skills at all three time points, however, females demonstrated a larger improvement over time (Table 3).

### Education Levels

A significant time by group interaction was also found for education levels on practitioners’ self-rating of overall self-efficacy (Table 2) as well as their consultation skills self-efficacy (Table 3) at post training and at follow-up.

Practitioners with a doctorate or master’s degree rated their self-efficacy highest across all three time points (Table 1). However, the improvement rate from pre-training to post-training was largest among those with a bachelor degree (*M*_*change1*_ = 1.30, *SD* = 1.25) (Table 2). At follow-up, the improvement in practitioners’ overall self-efficacy was largest among those with a high school qualification (*M*_*change2*_ = 1.75, *SD* = 1.31). Similar findings were found for consultation skills self-efficacy with practitioners with a bachelor degree made the largest improvement at post training and high school/secondary education practitioners made the largest improvements at follow-up. Effect size of differences was 0.15 [0.14–0.16] at post training and 0.11 [0.11–0.13] at follow-up. (Table 3).

### Disciplines

A significant time by group interaction was found for discipline on practitioners’ self-rating of overall self-efficacy and consultation skills self-efficacy at both post training and follow-up (Table 2, Table 3). Post-hoc analysis revealed that both overall self-efficacy scores and consultation skills self-efficacy at pre-training, post-training, and follow-up were highest among mental health workers (Table 1). This group, however, had the smallest improvement scores across time points while allied professionals (Table 2) and medical practitioners had the largest improvement (Table 3).

### Country Groups

A significant time by group interaction was found for country groups on practitioners’ overall self-efficacy (Table 2) and consultation skills self-efficacy (Table 3) at post training and follow-up. For country groups, English-speaking practitioners reported a larger improvement in their Self-efficacy than non-English-speaking practitioners. The change in practitioners’ overall self-efficacy was larger in English speaking group at post-training (*M*_*change1*_ = 1.35, *SD* = 1.23), and from pre-training to follow-up (*M*_*change2*_ = 1.79, *SD* = 1.31) comparing to (*M*_*change1*_ = 082, *SD* = 1.07) and (*M*_*change2*_ = 1.23, *SD* = 1.14) within the non-English speaking practitioners. The improvement of practitioners’ consultation skills self-efficacy was also larger among English speaking compared to non-English-speaking countries at both post training and follow-up (Table 3). The effect size of difference in term of their improvement in consultation skills was 0.16 [0.14–0.18] at post training and 0.13 [0.12–0.15] at follow-up.

### Study 2

#### Overall training outcomes

Outcomes indicated that practitioners’ overall self-efficacy improved significantly at post training (*F*(1, 4120) = 6151.92, *p* < 0.0001) and at follow-up (*F*(1, 4120) = 9336.50, *p* < 0.0001). Their consultation skills self-efficacy also significantly improved from pre-training to post training (*F* (1, 4120) = 11740.14, *p* < 0.0001) and to follow-up (*F* (1, 4120) = 14456.73, *p* < 0.0001) (Table 4).

**Table 4:** Study 2 means and standard deviations of PCSC subscales at pre-training, post-training, and follow-up by moderators

Variable			Practitioner confidence						Consultation skills					
			Pre		Post		Follow-up		Pre		Post		Follow-up	
	*n*	*%*	*M*	*SD*	*M*	*SD*	*M*	*SD*	*M*	*SD*	*M*	*SD*	*M*	*SD*
*Overall training outcomes*	6867		4.38	1.31	5.72	0.86	6.12	0.75	4.55	1.13	5.80	0.76	6.11	0.64
*Gender*														
Female	6057		4.35	1.31	5.71	0.86	6.12	0.76	4.53	1.13	5.80	0.75	6.11	0.64
Male	665		4.70	1.30	5.85	0.84	6.18	0.76	4.74	1.17	5.84	0.79	6.15	0.66
*Education levels*														
Bachelor	2945		4.41	1.32	5.77	0.86	6.15	0.74	4.61	1.13	5.85	0.74	6.15	0.62
Doctorate	106		4.74	1.34	6.03	0.84	6.24	0.75	4.71	1.17	6.07	0.68	6.21	0.68
Master	1725		4.47	1.36	5.78	0.87	6.15	0.74	4.63	1.08	5.83	0.74	6.13	0.62
Highschool	336		4.14	1.30	5.49	0.90	6.06	0.80	4.28	1.27	5.62	0.88	6.08	0.73
Tertiary^a^	1143		4.35	1.27	5.68	0.85	6.10	0.77	4.46	1.15	5.72	0.78	6.05	0.66
*Disciplines*														
Allied Health and Correction Services	216		4.26	1.33	5.60	0.84	6.05	0.76	4.38	1.16	5.68	0.75	6.05	0.63
Teachers/Educators	1313		4.37	1.29	5.79	0.87	6.20	0.70	4.55	1.12	5.84	0.75	6.15	0.61
Mental Health Worker^b^	2937		4.56	1.30	5.80	0.84	6.17	0.73	4.73	1.07	5.88	0.72	6.17	0.61
Medical Personnel	433		3.85	1.18	5.39	0.84	5.79	0.79	3.92	1.08	5.41	0.75	5.75	0.71
Others	1282		4.31	1.38	5.73	0.88	6.18	0.74	4.51	1.21	5.82	0.76	6.15	0.62
*Training mode*														
In person	5183		4.38	1.29	5.73	0.85	6.12	0.75	4.56	1.10	5.80	0.74	6.11	0.63
Videoconference	1684		4.37	1.38	5.69	0.87	6.11	0.76	4.52	1.21	5.78	0.81	6.12	0.65

^a^Tertiary includes Trade/Diploma

^b^Mental Health Worker included Psychologists, Social Workers and Counselors

#### Training outcomes by training mode

Robust mixed ANOVAs found no significant interaction between time and mode of training, indicating that training outcomes were similar between in-person and videoconference training (*F* (2, 4477.325) = 1.752, *p* > 0.0001). Robust mixed ANOVAs were then conducted for the videoconference training sample to determine if potential moderators of gender, level of education, and discipline impact training outcomes within the online system. A significant interaction effect was found for practitioners’ gender for their overall self-efficacy (*F* (1, 1122.01) = 13.15, *p* < 0.0001) at follow-up and significant interaction effect for practitioners’ consultation skills self-efficacy at both post training (F(1, 1070.20 = 12.25, *p* < 0.0001) and at follow-up (*F* (1, 1109.96) = 19.26, *p* < 0.0001). This finding indicated that female practitioners reported higher rate of improvement of overall self-efficacy (at follow-up) and consultation skill self-efficacy (at both post training and at follow-up) compared to their male counterparts. In term of practitioners’ discipline, significant interaction was also found for consultation skill efficacy (but not overall self-efficacy) at post training (*F* (4 123.6218) = 5.76, *p* < 0.0001) and at follow-up (*F*(4 122.34) = 7.17, *p* < 0.0001). Change in practitioners’ consultation skills self-efficacy was observed to be largest among allied professional and medical personnel (Table 4).

## Discussion

This study examining the change in practitioners’ self-efficacy attended an international professional training program designed for a multidisciplinary workforce to deliver evidence-based parenting support. Consistent with our hypothesis, participation in Triple P training was associated with improvements (both short and long term) in practitioners’ self-efficacy to deliver the Triple P program with parents. These changes are consistent with the theoretical basis of the Triple P training program, which strongly emphasizes the importance of strengthening practitioners’ self-regulatory skills, (Mazzucchelli & Ralph, 2019). These finding are also consistent with previous studies (Ralph & Dittman, 2018; Sanders et al., 2003; Sethi et al., 2014) where practitioners participating in the training program typically reported an improvement of their self-efficacy regardless of their disciplines. By becoming self-efficacious, practitioners can tailor the program to provide parents with suitable support and create a structural environment that encourages and activates parents’ self-regulatory capacity. Parents, in turn, learn to create a nurturing and responsive environment in their home that contributes to the promotion of self-regulation in children (Turner et al., 2011).

When examining the effects of different practitioners’ and program factors on training outcomes, we found that there were significant differences in practitioners’ reports across genders, with male practitioners rating their self-efficacy higher than females at each time point, but female practitioners improved at a larger rate than their male counterparts. This finding is consistent with the self-efficacy literature suggesting differences across gender. Previous studies of self-efficacy across learning contexts have shown that females are more likely to apply self-management strategies in their learning, and thus display more outstanding learning outcomes than male counterparts. However, females tend to be more modest in their self-reports and rate themselves lower than males who tend to overrate their confidence in skills they may or may not possess (Pajares, 2002). The differences across groups in this current study were nevertheless small and indicated minimal clinical impact.

Among different practitioners’ factors, educational levels and their cultural-language training context seems to have the largest impact on training outcomes. To be more specific, practitioners with a post-graduate degree rated themselves higher at each time point but those with high school/bachelor’s degree showed the largest improvement. This is not altogether surprising as the more unfamiliar the training might have been to practitioners, the greater room for them to improve. In term of cultural-language background, we found that although both English-speaking and Non-English-speaking practitioner’s indicated similar level of overall self-efficacy post training; Non-English speaking practitioners demonstrated smaller improvement in their specific consultation skills self-efficacy over time.

This finding can be explained in several ways. First, though local trainers speaking local languages were employed when possible, most training programs at Triple P are delivered in English. Non- English speaking practitioners thus, may find difficult to comprehend the content, resulting in a lack of confidence. Second, different cultures might hold different views about parenting and intervention. As Turner et al. (2011) specified, training effectiveness in promoting self-efficacy may vary depending on practitioner characteristics, such as practitioners’ cultural orientation, their beliefs, the complexity of the families they work with, their knowledge of the evidence-based intervention, and their belief that the program is effective. The Triple P program - initially developed in Australia –a developed and individualistic culture- may holds values and beliefs that may not fit the values and beliefs of practitioners from other linguistic and cultural backgrounds. Additionally, non-English-speaking practitioners may have limited access to evidence-based parenting resources, mostly written in English, which may affect their motivation and perception to engage deeply with the program (Weisz & Gray, 2008). This may have an impact on the training outcomes and practitioners’ ability to make significant improvements during and after training. This interpretation of the outcomes however should be taken with care given the small effect size found. Further studies are warranted to understand the impact of other practitioners’ cognitive factors on training outcomes, including their satisfaction with training, practitioners’ belief in the program’s values and their perception of whether the program is a good fit for their clients.

The results of our study showed that there were no significant differences between online and in-person training. This finding is particularly noteworthy given the use of online training in clinical professional training is still relatively new. This result can be attributed to a number of reasons. First, while online learning might offer less opportunities for active practicing than face-to-face learning, it might facilitate other learning strategies that ultimately will enhance self-efficacy. Previous studies have found that online courses’ structure and content indeed, promote self-paced learning (Chiong, 2009; Vrasidas & McIsaac, 2000). In Triple P, all training courses last between two and five days, and practitioners are not accredited until six weeks post training. A short training period and a long interval between training and accreditation may have given practitioners time to consolidate their skills and absorb course content at a pace that suits them. In addition to that, research has also found that online environments can actually encourage students’ intrinsic motivation through their increased sense of autonomy (Baru et al., 2020; Wadsworth et al., 2007), thus support the development of self-efficacy. As a result, participants taking online courses may be more committed to the learning process and may therefore put forth the additional effort required to succeed and might feel more confident about their abilities to work with clients in the future.

We must also note, however, that we only relied on self-reported data in our study. Previous studies suggested that individuals might overestimate their abilities when they do not have specific skill demonstrations upon which to assess their abilities (Davis et al., 2006). Practitioners of online training who participated in videoconferencing might have overestimated their self-efficacy due to a lack of experience or feedback to validate their perceptions. An objective measure of competency would be helpful in future studies to provide a more comprehensive understanding of the training outcomes. Additionally, future studies should continue to investigate how moderators could affect training outcomes in online environments. Our study found that only practitioners’ gender and discipline affected training outcomes in the videoconference training modality, contrary to Study 1. However, the findings should be interpreted with caution and further validated, as differences in sample sizes between two studies may have influenced the outcomes.

### Limitations and Implications

Our study used routinely collected pre-post data available as part of the on-going training approach used in the global dissemination of Triple P system. This is the first large-scale study that examines the change of practitioners’ self-efficacy over the course of Triple P professional training system under usual practice conditions. This study shows that an international professional training system can be delivered in diverse cultures and real-world contexts to develop practitioners’ confidence and efficacy in delivering evidence-based parenting support. However, there are limitations to this study that need to be addressed. First, the quasi-experimental design without controlled arm(s) precludes our ability to make causal inferences between training and practitioners’ outcomes. However, in real-world situations, randomized controlled trials with alternative arm(s) are not always possible. Even, when possible, its design restrictions do not always provide an accurate measure of program effectiveness in the usual context of practice.

In this study, we primarily interested in the improvement of practitioners’ self-efficacy, thus findings were based exclusively on practitioner report. The self- administered PCSC though was found to be reliable needs to be further assessed and evaluated for construct validity and predictive validity, ensuring that the scale fit and is valid for the purpose of capturing changes over time. It is important to note that changes in therapists’ self-efficacy do not necessarily concord with change in therapist’s actual competency (Beidas et al., 2009; Herschell et al., 2009). Future training studies would benefit from additional observational measures similar to those used by Sanders et al. (2020). Videotaped recordings of actual sessions were coded using a reliable observational measure designed to assess both content and process fidelity. This involved assessing coverage of the required session content (activities) and the quality of delivery of the session (Sanders et al., 2020).

Although the Triple P professional training system appears to be successful in promoting practitioners’ self-efficacy to begin implementing evidence-based programs, the continuous delivery of the program is influenced by other range of organizational factors beyond the quality of their initial training (Côté & Gagné, 2020; Sanders & Murphy‐Brennan, 2010; Seng et al., 2006). Future longitudinal research that incorporates an analysis of organizational factors, as well as training factors, would provide valuable insight into the role of professional training with other implementation strategies as part of the implementation process. Other aspects of self-regulation should also be examined in future studies such as self-management skills (goal setting, self-monitoring, and self-evaluation), personal agency (attributing change to one’s own effort), self-sufficiency (becoming self-reliant) and problem-solving (applying principles and strategies to solve new/different consultation problems). As professional learning is a long term process, the kinds of reflective practices encompassed by a self-regulation approach may have other benefits such as continuous self-improvement in the use of evidence-based programs, openness to peer feedback through the PASS model of supervision (Peer Assisted Supervision and Support) (McPherson & Schroeter, 2018; Sanders, 2012), and implementation strategies designed to support agencies embedded within routine services (McWilliam et al., 2016; Roppolo et al., 2019).

While there is growing evidence of Triple P’s cultural robustness, it is essential to highlight that parents’ perception of the program’s value might not accurately reflect practitioners’ perception of the program and vice versa. Studies that assessed Triple P’s cultural acceptability showed that parents from diverse cultural backgrounds rate Triple P content and materials more favorably, than practitioners’ working with cultural diverse parents. Parents of diverse cultural backgrounds also identified fewer barriers to use the program than did practitioners (Morawska et al., 2012). These findings, together with the significant difference between English and non-English-speaking practitioners’ self-efficacy improvement found in this study, suggested that further examination is required to understand the possible physical, perceptional, and contextual barriers that may hinder non-English-speaking practitioners’ self-efficacy and the type of support needed for non-English-speaking practitioners during and after training. It would be interesting to explore the link between practitioners’ program satisfaction and program outcomes (including both practitioners’ self-efficacy and the accreditation rate) while controlling for other practitioner characteristics. Such study will provide insights into the independent impact of each variable and how they might interact within the same context to impact training outcomes.

Another important training consideration is accessibility. The current training model was based on in-person training being provided by one trainer to a maximum of 20 practitioners. A challenge arises, particularly in smaller organizations or regions new to Triple P, where a smaller number of staff require training. This can result in delays or additional costs for practitioner travel to attend training at a central location. The finding that videoconference training achieved similar outcomes to in-person training creates the possibility of continuing with this delivery modality, which will increase access to training for many practitioners, particularly those in regional and remote areas. With the COVID-19 pandemic making in-person training very difficult, videoconference training became essential. However, videoconferencing is not a panacea and some agencies initially struggled to ensure their IT systems could accommodate Zoom technology. Practitioners themselves had to become used to interacting with peers and trainers remotely and confident in using the technology. All practitioners needed suitable cameras, microphones and speakers and had to learn a new etiquette relating to equipment use (e.g., muting unless speaking, always keeping the video on). Many practitioners after months of videoconferences and telehealth delivery to clients reported “zoom” fatigue. Notwithstanding these challenges, this study showed the majority of practitioners rated their videoconference-based training very favorably. Overall, these findings are encouraging and highlight the feasibility of delivering at scale a professional training course on evidence-based parenting support in a global context.
